# Risk of iatrogenic pneumothorax based on location of transbronchial biopsy: a retrospective cohort study

**DOI:** 10.1186/s13104-023-06275-5

**Published:** 2023-02-13

**Authors:** Mina Ishak, Debarati Chakraborty, Shayan Kassirian, Inderdeep Dhaliwal, Michael A. Mitchell

**Affiliations:** Department of Medicine, Schulich School of Medicine and Dentistry, Western University, London, ON Canada

**Keywords:** Pneumothorax, Transbronchial biopsy, Bronchoscopy

## Abstract

**Objective:**

Transbronchial lung biopsy (TBB) is a commonly performed procedure to obtain parenchymal lung tissue during bronchoscopy. Pneumothorax is among the most common serious complications of TBB. The objective of this study was to assess whether location of TBB correlated with development of post-procedural pneumothorax. We also sought to identify additional risk factors associated with pneumothorax development.

This was a single-centre, retrospective cohort study. All TBB performed between 2010 and 2020 underwent subsequent chart review. The primary outcome was radiologist reported pneumothorax on post-procedure chest x-ray. Multivariable logistic regression model was created with included variables chosen a priori based on clinical significance.

**Results:**

There were a total of 222 TBB performed that met inclusion criteria. Radiographic evidence of pneumothorax was reported in 38 patients (15.4%). Ten patients (4.1%) required a chest tube. In the multivariable analysis, risk of pneumothorax was significantly higher for biopsies obtained from the left upper lobe (OR 3.3; 95% CI 1.3–9.1). There was an increased risk of pneumothorax following TBB when obtained from the left upper lobe. Clinicians should be aware of the increased risk and should consider alternative locations in patients with diffuse lung disease.

**Supplementary Information:**

The online version contains supplementary material available at 10.1186/s13104-023-06275-5.

## Introduction

Transbronchial biopsy (TBB) is a commonly performed procedure during flexible bronchoscopy to obtain alveolar tissue for pathologic examination [1]. It is useful in a variety of clinical scenarios including in interstitial lung diseases such as a sarcoidosis and hypersensitivity pneumonitis, or to sample a peripheral lung lesion [2, 3]. It can be performed without real-time image guidance or with the use fluoroscopic or other guidance methods, depending on availability and centre-specific practices [4].

The two most common complications of TBB are bleeding and pneumothorax, with the later having a reported incidence of 1–6% [5]. Risk factors for the develop of pneumothorax from TBB include age, number of biopsy specimens obtained, and emphysema [6–8]. Interestingly, there is a purported association between location of TBB and risk of pneumothorax. A 2019 study by Herout et al. specifically addressed this question in a retrospective analysis. They concluded that the risk of post-procedure pneumothorax was significantly increased when TBB was performed from the left upper lobe (LUL) and recommended to avoid TBB from the upper lobes in diffuse lung disease [1]. It was hypothesized that the apical-basal intrapleural pressure gradient may be a possible explanation for this finding. These results have not been subsequently replicated. As such, the objective of this study was to assess whether location of TBB correlated with development of post-procedural pneumothorax in our population.

## Main text

### Materials and methods

This was a retrospective cohort study at an academic, tertiary-care centre in Ontario, Canada. This study was approved by the research ethics board at Western University (REB#115469). All adult (age ≥ 18) transbronchial biopsies specimens obtained between January 1st, 2010, and December 31st, 2020 were identified by the Pathology Research Service. Chart reviews were subsequently completed (by D. C.) to document patient demographics, location (inpatient vs. outpatient) and clinical variables including indication for TBB, number of biopsies obtained as well as location of TBB (by lobe). Post procedure chest x-rays reports were reviewed to determine if a pneumothorax had occurred, which was based on the interpretation of the reporting radiologist. It is routine to obtain an upright portable chest x-ray post procedure after performing TBB at our centre. All chest x-rays within 24 h of procedure were reviewed. Charts were also reviewed to determine if patients had developed a delayed pneumothorax. If a pneumothorax was present, the need for chest-tube placement was documented. Patients who did not have a post procedure x-ray were excluded as we could not definitively determine whether a pneumothorax was present.

At our centre, all bronchoscopies are performed or supervised by a respirologist or thoracic surgeon. Trainees, most commonly post-graduate years 4 and 5, are typically present and participate in the bronchoscopy and performance of TBB. Bronchoscopies requiring TBB are typically performed in the endoscopy suite under conscious sedation. TBB were obtained using a flexible bronchoscope, without fluoroscopic guidance, using 2 mm biopsy forceps. TBB performed under the guidance of radial-probe endobronchial ultrasound were excluded due to the marked difference in biopsy technique (see Additional file 1).

### Data analysis

Statistical analyses were completed using SAS version 9.4 (SAS Institute). Missing data was not imputed. The characteristics of the study cohort were summarized as mean (standard deviation [SD]) for normally distributed data and median (interquartile range [IQR]) for non-normally distributed data. Our primary outcome was post-procedure pneumothorax. Univariable logistic regression was performed to calculate odds ratios (OR) with 95% confidence intervals [CI]) for each variable. Multivariable logistic regression was performed for the primary outcome of post-procedure pneumothorax using variables chosen a priori based on clinical importance to calculate OR with 95% CI.

## Results

A total of 246 TBB were obtained, with each being performed in a unique patient. Twenty-four (10%) patients were excluded as they did not have a post-procedure chest x-ray (Table 1). A total of 222 patients were included in the analysis. Patient demographics and procedure-related data are listed in Table 1. The mean (± SD) age was 62 (± 15) with 49% being female. Median (IQR) BMI was 27 (23–31). Most procedures were performed on outpatients (64%). Suspected atypical infection was the most common indication for transbronchial biopsy (43%) followed by suspected malignancy (31%) and interstitial lung disease (10%). Fifty percent of patients had 5 or more biopsy specimens obtained while the other 50% had 4 or fewer. The most common location for TBB was the right lower lobe (38%), followed by the right upper lobe (18%), left lower lobe (17%), left upper lobe (17%) and right middle lobe (11%).

**Table 1:** Characteristics of study cohort (n = 246)

Age (mean)	61.8 (SD ± 15.3) years
Sex	
Male	114 (51.4%)
Female	108 (48.6%)
Patient Location	
Outpatient	140, (64.2%)
Inpatient	78 (35.8%)
BMI (median)	26.5 (IQR 23.1–30.6)
Indication for biopsy	
Suspected interstitial lung disease	23 (10.4%)
Suspected atypical infection	96 (43.2%)
Suspected malignancy	68 (30.6%)
Other	35 (15.8%)
Location of biopsy	
Right lower lobe	85 (38.5%)
Right middle lobe	26 (11.8%)
Right upper lobe	39 (17.6%)
Left lower lobe	36 (16.3%)
Left upper lobe	35 (15.8%)
Number of biopsies obtained	
1–4	112 (50.4%)
> = 5	110 (49.6%)
Pneumothorax	38 (15.4%)
Pneumothorax requiring chest tube	10 (4.1%)

*BMI* body mass index (Kg/m^2^), *SD* standard deviation, *IQR* interquartile range

There was a total of 38 (17%) radiologist reported pneumothoraces on post-procedure chest x-ray. Seven (18%) of the pneumothoraces were described as ‘questionable’ by reporting radiologists and did increase in size on repeat imaging. There were no delayed presentations of pneumothorax requiring hospitalization. A comparison between patient groups with or without radiological evidence of pneumothorax is shown in Table 2. Of the 38 patients who had a reported pneumothorax, 10 (4.1%) required the insertion of a chest tube as part of their management. In the multivariable analysis, the odds of pneumothorax were significantly higher for TBB obtained from the left upper lobe (OR 3.3; 95% CI 1.3–9.1). No other biopsy locations were significantly associated with risk of pneumothorax. Inpatient status at the time of the procedure was also significantly associated with increased risk of developing a pneumothorax (OR 2.6, 95% CI 1.2–5.6). Having a BMI > 30 (OR 0.7, 95% CI 0.3–1.7) or obtaining 5 or more biopsies (OR 0.6, 95% CI 0.3–1.2) were not significantly associated with pneumothorax (see Table 3).

**Table 2:** Comparison of patients with and without pneumothorax

Variable	Pneumothorax (n = 38)	No pneumothorax (n = 184)	^a^OR (95% CI)
Sex			
Male	21 (18.4%)	93 (81.5%)	[ref]
Female	17 (15.7%)	91 (84.3%)	0.80 (0.40–1.62)
BMI			
< = 30	30 (18.1%)	136 (81.9%)	[ref]
> 30	8 (14.3%)	48 (85.7%)	0.76 (0.32–1.86)
Number of biopsies obtained			
1–4	23 (20.5%)	89 (79.5%)	[ref]
> = 5	15 (13.9%)	93 (86.1%)	0.63 (0.31–1.27)
Patient location			
Outpatient	18 (12.9%)	122 (87.1%)	[ref]
Inpatient	19 (24.4%)	59 (75.6%)	2.18 (1.07–4.46)
Location of biopsy			
Right lower lobe	11 (12.9%)	74 (87.1%)	[ref]
Right middle lobe	4 (15.4%)	22 (84.6%)	1.22 (0.35–4.22)
Right upper lobe	5 (12.8%)	34 (87.2%)	0.99 (0.31–3.07)
Left upper lobe	6 (16.7%)	30 (83.3%)	3.08 (1.19–8.01)
Left lower lobe	11 (31.4%)	24 (68.6%)	1.35 (0.45–3.97)
Indication for biopsy			
Suspected interstitial lung disease	4 (17.4%)	19 (82.6%)	[ref]
Suspected atypical infection	19 (19.8%)	77 (80.2%)	1.17 (0.36–3.85)
Suspected malignancy	10 (14.7%)	58 (85.4%)	0.82 (0.23–2.92)
Other	5 (14.3%)	30 (85.7%)	0.79 (0.19–3.33)

*OR* odds ratio, *CI* confidence interval

^a^Univariable logistic regression for primary outcome of post-procedure pneumothorax following transbronchial biopsy

**Table 3:** Multivariable logistic regression for primary outcome of post-procedure pneumothorax following transbronchial biopsy

Variable	OR (95% CI)
Location of biopsy	
Right lower lobe	[ref]
Right middle lobe	1.12 (0.30–4.12)
Right upper lobe	0.88 (0.27–2.86)
Left lower lobe	1.25 (0.40–3.90)
Left upper lobe	3.33 (1.25–9.06)
Number of biopsies obtained	
1–4	[ref]
> = 5	0.58 (0.27–1.24)
Patient location	
Outpatient	[ref]
Inpatient	2.61 (1.22–5.58)
BMI	
< = 30	[ref]
> 30	0.66 (0.26–1.69)

*BMI* body mass index (Kg/m^2^), *OR* odds ratio, *CI* confidence interval

Finally, 80 of the 246 (32.5%) patients in our study had pathology results from their transbronchial biopsies which yielded a diagnosis. The diagnoses from the pathology varied between malignancy, granulomatous disease (with the majority being sarcoidosis), and organizing phenomena of different aetiologies.

## Discussion

In our study, there was a significant association between TBB obtained from left upper lobe and development of post-procedure pneumothorax. Our results are consistent with the study previously published by Herout et al., which also found a significantly higher risk of pneumothorax in the left upper lobe (1). Interestingly, in both studies there was also a trend towards increased risk of pneumothorax following TBB from the right upper lobe, however, this was not found to be statistically significant. Additionally, in a study by Huang et al. 10 out of 13 post-TBB pneumothoraces occurred from biopsy of the upper lobes, although this study was not adequately powered to show a significant difference between lobes.

The rationale for the increased risk of pneumothorax post TBB obtained from the upper lobes is not well understood. Subpleural blebs and emphysema are more prominent in the upper lobes, which may predispose to pneumothorax if inadvertently biopsied. In addition, alveoli at the apices of the lung are more distended and less compliant than the bases because of the pleural pressure gradient [9]. The effect of gravity and the weight of the lung may also play a role; a tear in the dependent visceral pleura may be sealed due to the weight of the lung, particularly when the patient remains upright after the procedure If a defect occurs in the apical lung, the weight of the lung may promote recoil away from the chest wall, causing a pneumothorax to accumulate. The true mechanism for this phenomenon however remains uncertain.

It is also unclear why there are differences in pneumothorax between the right and left upper lobes. The anatomy of the bronchial tree may provide a possible explanation. In the left upper lobe, the apical-posterior and anterior segments branch at a broad angle in relation to the lingula. A pneumothorax in the apical-posterior or anterior segments could promote recoil downward and laterally towards the lingular segment. For comparison, in the right upper lobe, only the apical segment exits at a broad angle and would have the tendency to recoil downward. In addition, difficulty in correctly judging the appropriate depth of biopsy in the apical-posterior segment of the left upper lobe may explain why there is an increase in risk of pneumothorax, although this is purely speculative.

We found that the patients admitted to hospital at the time of their procedure had a higher risk of pneumothorax which is a novel finding not previously reported. More severe underlying lung disease as well as acute presentations of lung disease are inherent to the inpatient population and may account for this finding. Previous studies that examined the rates of complications from bronchoscopy in the outpatient setting often reported lower rates than that in the inpatient setting [13, 15]. Clinicians should be aware of this increased risk and take it into consideration when determining if to proceed with TBB for their inpatient population.

The incidence of pneumothorax in our study is higher than most previously published studies [1, 5, 10, 12]. One reason to explain this discrepancy may be related to the outcome used in our study of radiologist reported pneumothorax. Seven of the pneumothoraces included were reported as questionable and were of no clinical significance. Moreover, others were small and clinically insignificant. Only 4.5% of individuals developed pneumothorax that required intervention with a chest tube which is consistent with the established incidence of 1–6% [5, 12]. Additionally, 24 patients were not included in the analysis as they did not have a post-procedure chest x-ray. It is unlikely these individuals had a symptomatic pneumothorax given lack of imaging requirement. As such, the 17% risk of pneumothorax may be an overestimate due to lack of inclusion of these individuals. Lastly, operator experience may influence the complication rate. Our study was conducted at a teaching center with differing levels of operator experience. In a large single-cohort study, Yeow et al. reported operator experience as the third major risk factor for pneumothorax in CT guided lung biopsies [11], although this has not been reliably found to be associated with increased risk of pneumothorax [8, 14].

In conclusion, our study found that the risk of pneumothorax was highest when TBB was performed from the left upper lobe. Clinicians should be aware of this increased risk and take it into consideration when determining preferred location of biopsy for individuals with diffuse lung disease. Further evidence, ideally a prospective multi-centre study, is needed to confirm this finding.

## Limitations

Our study has several limitations which should be considered when interpreting the results. Firstly, it is a single-centre study and therefore generalizability of our results may be limited. Transbronchial biopsy technique was not standardized and thus varied between operators. Our results, however, are largely consistent with those previously published by Herout et al. (1). Next, all procedures in our study were performed without the use of fluoroscopy. Indication for blind transbronchial biopsy is becoming increasingly scarce with the uptake of fluoroscopy and endobronchial ultrasound guidance, however previous data suggests that while fluoroscopy may have a higher diagnostic yield than a blind approach, it does not reduce the risk of pneumothorax [16]. Nonetheless, our findings are not generalizable to centres performing TBB guided by fluoroscopy. In addition, our study did not examine the patient’s underlying lung disease and whether that rates of pneumothorax were higher in specific groups. For instance, emphysema has been associated with higher risk of pneumothorax in other multivariate analysis [6, 17]. However in our study we were not able to adjust for underlying lung disease and assess the effects on the rates of pneumothoraxes. Lastly, as this was a retrospective study, causality between location of TBB and risk of pneumothorax cannot be determined as selection bias may be present.

## Supplementary Information

**Additional file 1.** Response to Reviewer Comments.

## Data Availability

All data generated or analysed during this study are included in this published article [and its Additional files].

## Abbreviations

TBB: Transbronchial biopsy
LUL: Left upper lobe
