# Supplementary material for: Risk of iatrogenic pneumothorax based on location of transbronchial biopsy: a retrospective cohort study
Source: BMC Res Notes. 2023 Feb 13;16:14. doi: 10.1186/s13104-023-06275-5 (PMC9926766; doi:10.1186/s13104-023-06275-5)
Supplement: Supplementary file 1 — Additional file 1. Response to Reviewer Comments. [file 13104_2023_6275_MOESM1_ESM.docx]

1. **In the current clinical situations, transbronchial lung biopsy for lung cancer should be performed under the fluorescent guidance or ultrasound guidance because of urgent need of obtaining enough tumor tissues for molecular analysis. Thus, bronchoscopic biopsy for peripheral lung tumor without any guidance may be powerless and uninteresting for many bronchoscopists. Thus, authors should comment the usefulness or significance of performing TBB without any guidance.**

Thank you for your feedback. Certainly, there are quite a few centers that do not have access to endobronchial US-guided biopsies. Patients with suspected lung cancer should be referred to centers that have access to better techniques. Our study aims to look at the use of transbronchial biopsies as a whole, not only in lung cancer, which in our experience, is used frequently in settings of diagnostic uncertainty and to retrieve a biopsy in a more expedited fashion. However, we recognize its use is decreasing and will continue to with the uptake of EBUS. As such, we have included a point in our discussion in lines 175-179 “Indication for blind transbronchial biopsy is becoming increasingly scarce with the uptake of fluoroscopy and endobronchial ultrasound guidance, however previous data suggests that while fluoroscopy may have a higher diagnostic yield than a blind approach, it does not reduce the risk of pneumothorax.^16^”

[16] Smyth CM, Stead RJ. Survey of flexible fibreoptic bronchoscopy in the United Kingdom. Eur Respir J. 2002 Mar. 19 (3):458-63.

2. **Incidence of pneumothorax after TBB or TBLB may relate to fundamental lung disease such as pulmonary emphysema or fibrosis. Authors should evaluate how fundamental lung disease affected the incidence of pneumothorax in their study.**

Unfortunately, we do not have the data with regards to the patient’s pathological lung disease in this study. Emphysema has been associated with higher risk of pneumothorax in other multivariate analysis^1,,2^, however in our study we have not adjusted for underlying lung disease and as to whether this would at all effect the rates of pneumothoraxes.

[1] M.H. Tukey et al. Population-based estimates of transbronchial lung biopsy utilization and complications. Respir Med Volume 106, Issue 11, 2012: 1559-1565.

[2] Huang CT, Ruan SY, Liao WY, Kuo YW, Lin CY, Tsai YJ, et al. Risk factors of pneumothorax after endobronchial ultrasound-guided transbronchial biopsy for peripheral lung lesions. PLoS One. 2012;7:e49125. doi: 10.1371/journal.pone.0049125.

3. **How about the diagnostic rate of this study? I understand this study is focusing on the pneumothorax of TBB, however, the diagnostic yield of biopsy may be one of the most interesting subjects for many readers.**

Thank you for that feedback. We were able to go back and review every patient’s pathology results. A review of this data revealed that a total of 80 of the 246 patients in our study had pathology which yielded a diagnosis. That is 32.5% yield. These diagnoses varied between malignancy, granulomatous disease (with the majority being sarcoidosis), and organizing phenomena of different aetiologies.

4. I could not find Figure 1. But, is "Figure 1" necessary?

This was meant to state table 1 and not figure one, our apologies.
